# Acanthamoeba Keratitis With Herpetic Endotheliitis: A Rare Co-infection and Diagnostic Challenge

**DOI:** 10.7759/cureus.112793

**Published:** 2026-07-16

**Authors:** Chrysoula Sgourou, Georgios Bontzos, Anna Kouroupaki

**Affiliations:** 1 Ophthalmology, Korgialenio-Benakio Hellenic Red Cross Hospital, Athens, GRC

**Keywords:** acanthamoeba keratitis, co-infection, confocal microscopy, corticosteroids, herpetic endotheliitis

## Abstract

Acanthamoeba keratitis (AK) is a rare but potentially vision-threatening corneal infection that most commonly affects contact lens (CL) wearers and is frequently associated with exposure to water. The clinical presentation may mimic other infectious keratitides, particularly herpetic keratitis, thereby delaying diagnosis and appropriate treatment. We report a case of AK complicated by herpes simplex virus (HSV) endotheliitis, highlighting the diagnostic and therapeutic challenges of this uncommon co-infection. A 29-year-old woman presented with acute keratitis of the right eye after swimming while wearing CLs. Slit-lamp examination revealed a corneal epithelial defect with a stromal ring infiltrate suggestive of AK. Initial microbiological investigations were negative, but in vivo confocal microscopy demonstrated multiple hyperreflective cystic structures within the anterior stroma, consistent with Acanthamoeba cysts. Intensive anti-amoebic therapy with topical polyhexamethylene biguanide and chlorhexidine was initiated. Twelve days after treatment onset, the patient developed corneal edema and keratic precipitates with worsening vision, raising suspicion of viral endotheliitis. Polymerase chain reaction analysis of the aqueous humor was positive for HSV DNA, supporting the diagnosis of herpetic endotheliitis. Oral valaciclovir and topical corticosteroids were introduced while anti-amoebic therapy was continued. The patient showed gradual clinical improvement, with recovery of best-corrected visual acuity to 1.0 (20/20 Snellen) and resolution of corneal edema. At the six-month follow-up, no Acanthamoeba cysts were identified on confocal microscopy, and no recurrence was observed. This case shows the diagnostic complexity of AK, particularly when the clinical course is atypical or when co-infection occurs. It also highlights the importance of advanced diagnostic techniques, such as confocal microscopy and PCR, in guiding clinical management. Recognition of possible viral co-infection during the course of AK is essential, as appropriate adjustment of therapy can lead to favorable visual outcomes despite the potentially conflicting treatment strategies required for these two conditions.

## Introduction

Acanthamoeba is a free-living protozoan that can cause a rare but potentially vision-threatening corneal infection. The reported incidence of Acanthamoeba keratitis (AK) varies geographically, ranging from approximately one to 33 cases per million contact lens (CL) wearers annually. In contrast, herpes simplex keratitis remains one of the leading causes of infectious corneal blindness worldwide, with an estimated incidence of 10-30 cases per 100,000 persons per year. The most common risk factors include CL use and ocular trauma. Increased risk has also been reported in patients who swim while wearing CLs or use tap water for their storage and cleaning [1].

The initial clinical presentation of AK varies and is often misdiagnosed as herpetic keratitis. Patients may present with relatively mild ocular signs despite experiencing disproportionately severe ocular pain, a finding that is often considered suggestive of the disease. A high index of clinical suspicion is essential for early diagnosis and improved prognosis [2]. Additionally, advanced AK with ring infiltrates may occasionally mimic fungal or bacterial keratitis, further complicating the diagnostic process.

Several diagnostic techniques are currently available, including corneal scraping for culture and polymerase chain reaction (PCR). However, in vivo confocal microscopy (IVCM) has emerged as a valuable noninvasive diagnostic tool, demonstrating very high specificity (up to 100%) and high sensitivity (85.3-100%) [3]. Consequently, it is increasingly considered an important adjunctive modality in the early diagnosis of AK. Treatment typically involves topical anti-amoebic agents such as polyhexamethylene biguanide (PHMB) and chlorhexidine, often combined with broad-spectrum antibiotics, antifungals, and cycloplegic agents [4].

Herpes simplex virus (HSV), on the other hand, is a double-stranded DNA virus and a common cause of infectious keratitis worldwide. HSV can affect all layers of the cornea. Endothelial involvement may present as disciform, diffuse, or linear endotheliitis. Disciform keratitis is the most frequently observed form and typically presents as a round area of central stromal and endothelial edema. Additional clinical signs such as keratic precipitates (KPs), anterior chamber inflammation, and elevated intraocular pressure (IOP) may also be present. Diagnosis is primarily clinical and based on slit-lamp biomicroscopy, although aqueous humor PCR analysis may provide supportive evidence. Treatment generally involves topical corticosteroids combined with systemic antiviral therapy [5].

Although AK and HSV keratitis are well-recognized causes of corneal infection, their coexistence is rarely reported. Such co-infection can significantly complicate both diagnosis and management, particularly because corticosteroid therapy, which is indicated for HSV endotheliitis, remains controversial in AK [6].

To our knowledge, this is one of the few reported cases of AK complicated by HSV endotheliitis. This case highlights the importance of considering viral co-infection in patients with atypical clinical deterioration during AK treatment. It illustrates the challenges of balancing anti-amoebic therapy with corticosteroid therapy required for HSV endotheliitis.

## Case presentation

A 29-year-old woman who wore monthly soft CLs presented to our clinic with acute keratitis in the right eye. Three days before presentation, she had been prescribed topical fluoroquinolone drops by a general practitioner, but there was no clinical improvement. She reported swimming while wearing her CLs. There was no history of ocular trauma or systemic disease, and best-corrected visual acuity (BCVA) before the incident was 1.0 (20/20 Snellen).

**Figure 1:**
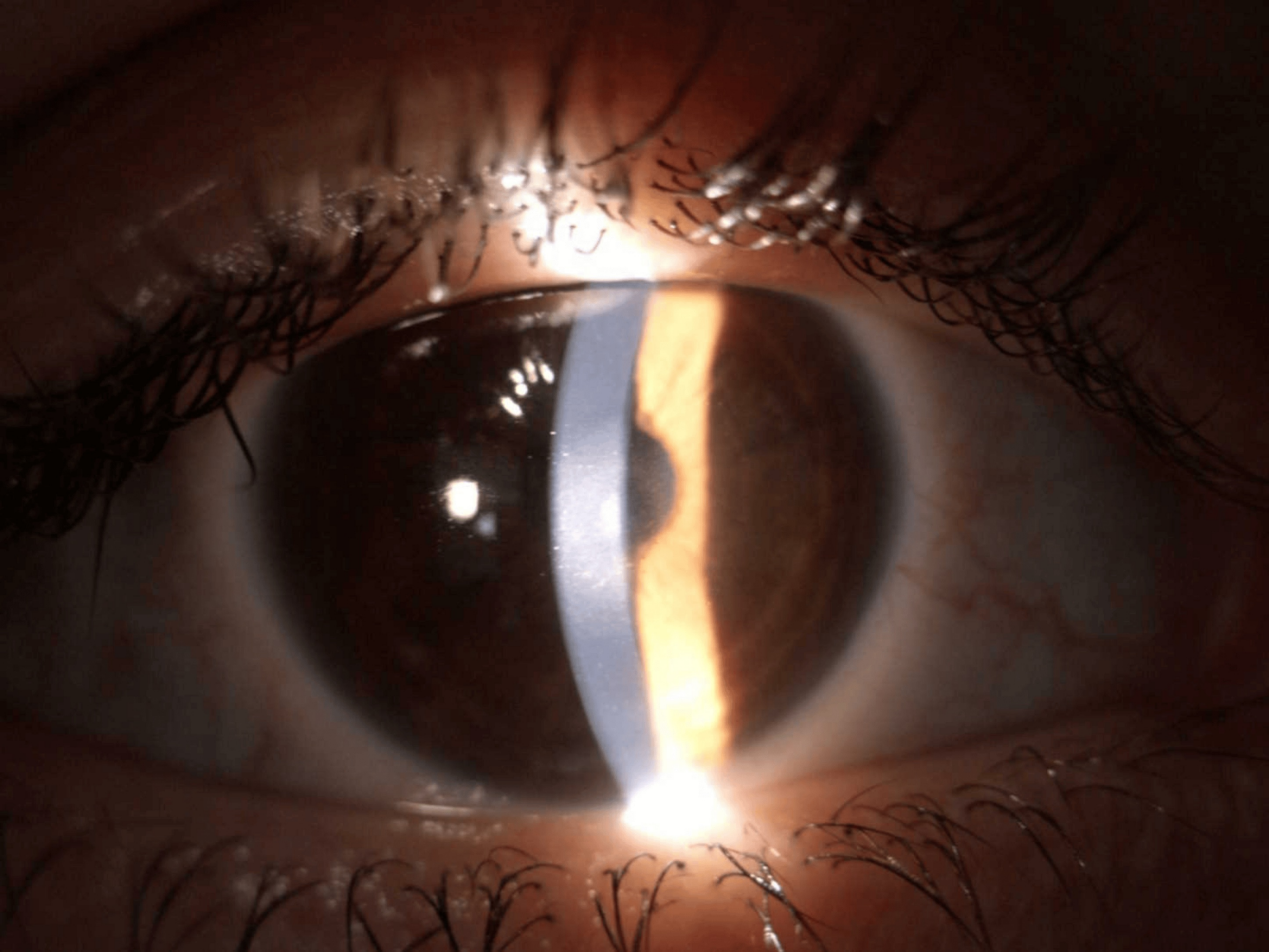
Slit-lamp photograph of the right eye at presentation demonstrating a large corneal epithelial defect with a characteristic ring-shaped stromal infiltrate

At presentation, BCVA, measured using decimal notation, was 0.2 in the right eye and 1.0 in the left eye. IOP was 15 mmHg in the right eye and 14 mmHg in the left eye. Slit-lamp examination of the right eye revealed a large corneal epithelial defect associated with a stromal ring-shaped infiltrate, findings suggestive of AK (Figure 1).

**Figure 2:**
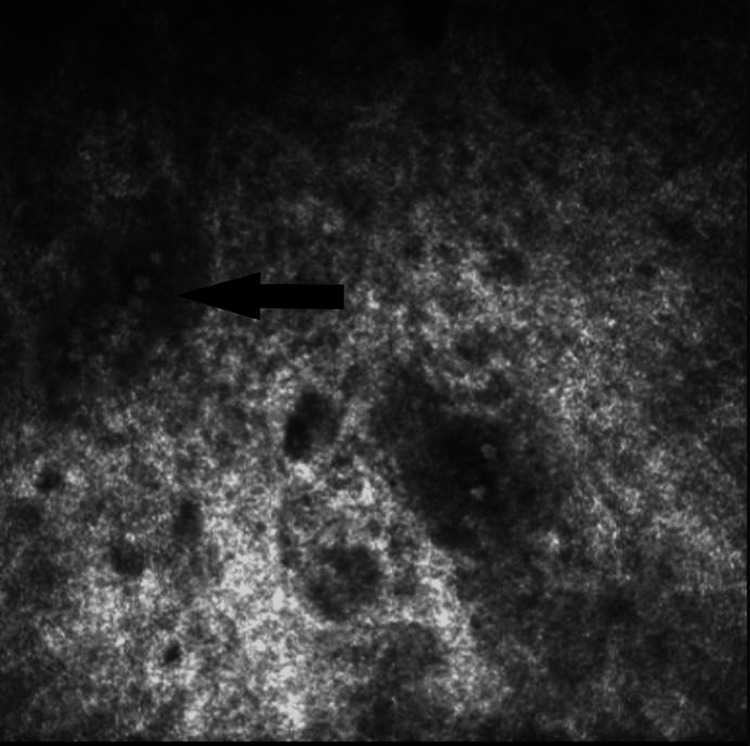
IVCM image demonstrating multiple hyperreflective, double-walled Acanthamoeba cysts (arrow) in the anterior corneal stroma at a depth of approximately 84 μm IVCM: in vivo confocal microscopy

The patient reported photophobia and tearing but did not describe severe ocular pain. Given the broad differential diagnosis of infectious keratitis, corneal scraping was performed for Gram staining and PCR analysis for HSV, varicella-zoster virus, cytomegalovirus, and Acanthamoeba. Initial microbiological testing was negative. Because of the characteristic clinical appearance and the patient’s contact-lens-related risk factors, IVCM was performed. Multiple hyperreflective cystic structures consistent with Acanthamoeba cysts were detected in the anterior stroma at a depth of approximately 84 μm, confirming the diagnosis (Figure 2).

The patient was admitted, and intensive anti-amoebic therapy was initiated with topical PHMB and chlorhexidine administered every 30 minutes, combined with topical voriconazole, fortified broad-spectrum antibiotics, and atropine twice daily. Systemic moxifloxacin 400 mg once daily was also started. Despite negative microbiological results, broad-spectrum antimicrobial therapy was continued empirically because previous antibiotic exposure may have reduced culture sensitivity and the extensive epithelial defect increased the risk of secondary bacterial infection. Over the following days, the epithelial defect gradually closed and visual acuity improved.

**Figure 3:**
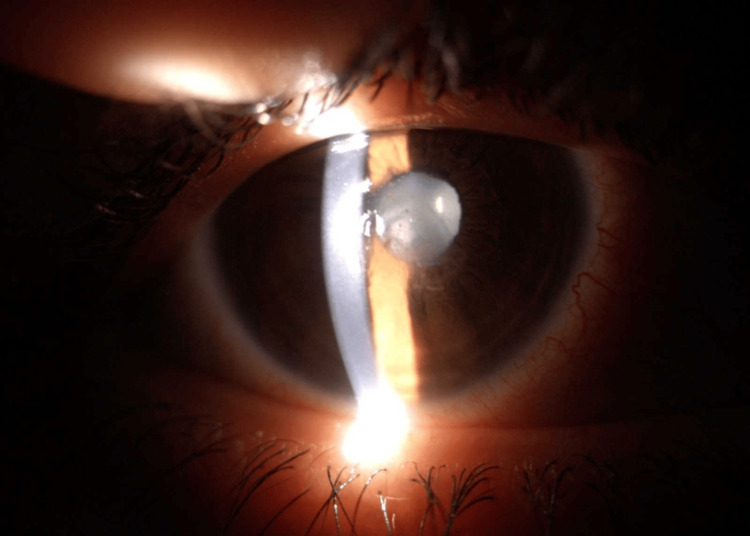
Slit-lamp photograph of the right eye demonstrating corneal stromal edema and newly developed KPs on the endothelium, consistent with herpetic endotheliitis KPs: keratic precipitates

However, 12 days after initiation of treatment, the patient developed new corneal edema and KPs, accompanied by a decline in BCVA from 0.5 to 0.1 (Figure 3).

**Figure 4:**
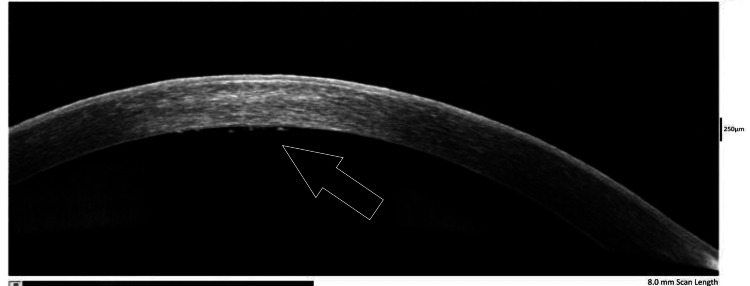
AS-OCT demonstrating stromal thickening and hyperreflective deposits (arrow) along the corneal endothelium, consistent with endothelial inflammation AS-OCT: anterior segment optical coherence tomography

These findings raised suspicion of secondary viral endotheliitis rather than progression of the Acanthamoeba infection. Anterior segment optical coherence tomography (AS-OCT) confirmed stromal edema and endothelial inflammatory deposits (Figure 4).

To clarify the diagnosis, aqueous humor sampling was performed, and PCR analysis detected HSV DNA, supporting the diagnosis of herpetic endotheliitis. Treatment was therefore modified to include oral valaciclovir 1000 mg three times daily and topical dexamethasone four times daily while anti-Acanthamoeba therapy was continued. During this period, IOP increased to 28 mmHg, and oral acetazolamide 250 mg twice daily was initiated. IOP normalized within 10 days, and acetazolamide was discontinued after two weeks.

**Figure 5:**
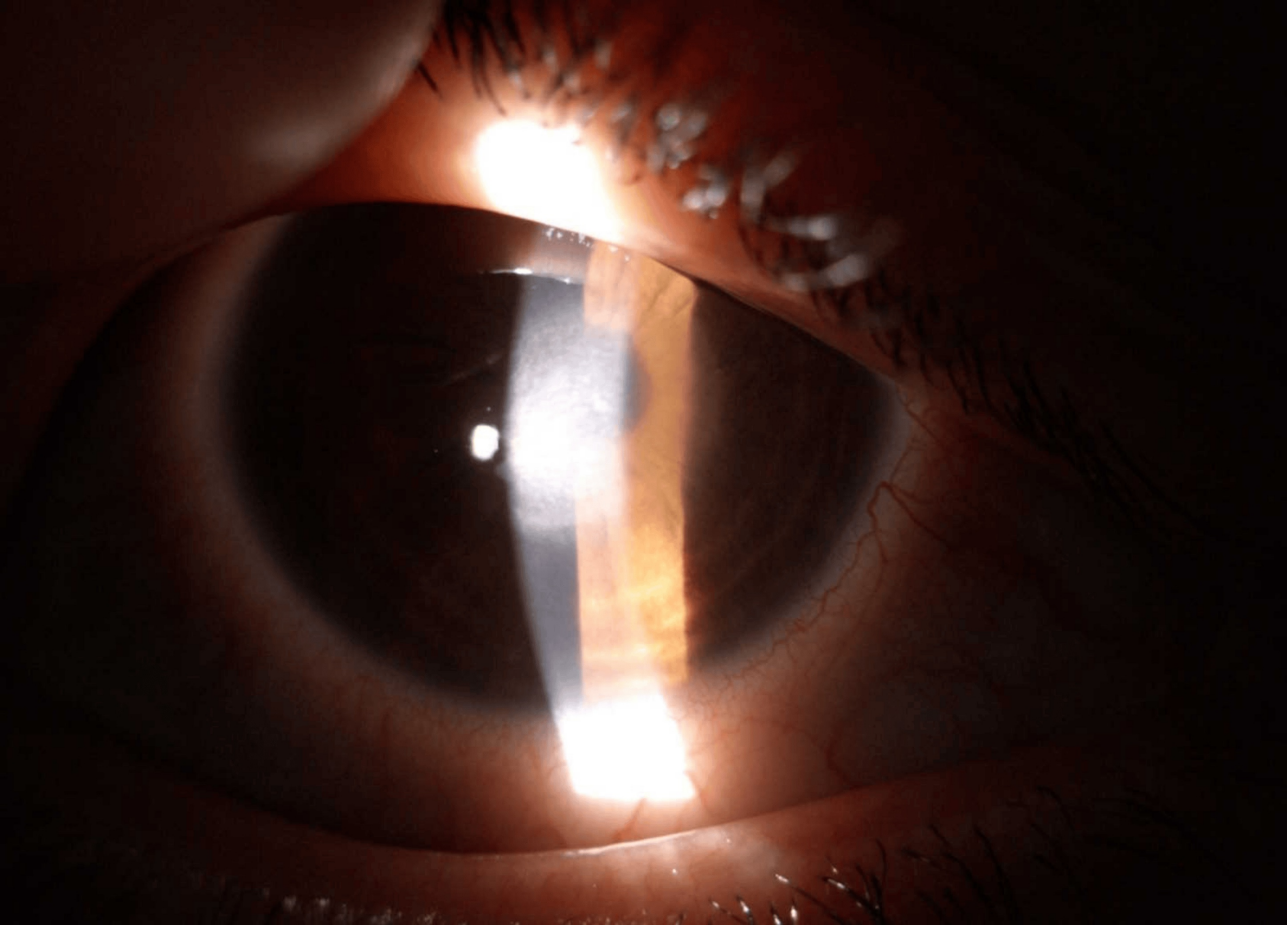
Slit-lamp photograph of the right eye at six-month follow-up showing mild stromal haze and superficial punctate keratitis, with no signs of active anterior chamber inflammation

Over the following months, the patient showed progressive clinical improvement. At the three-month follow-up, BCVA had improved to 1.0, and IOP had normalized. Slit-lamp examination showed mild stromal haze and superficial punctate keratitis, without evidence of active anterior chamber inflammation (Figure 5).

**Figure 6:**
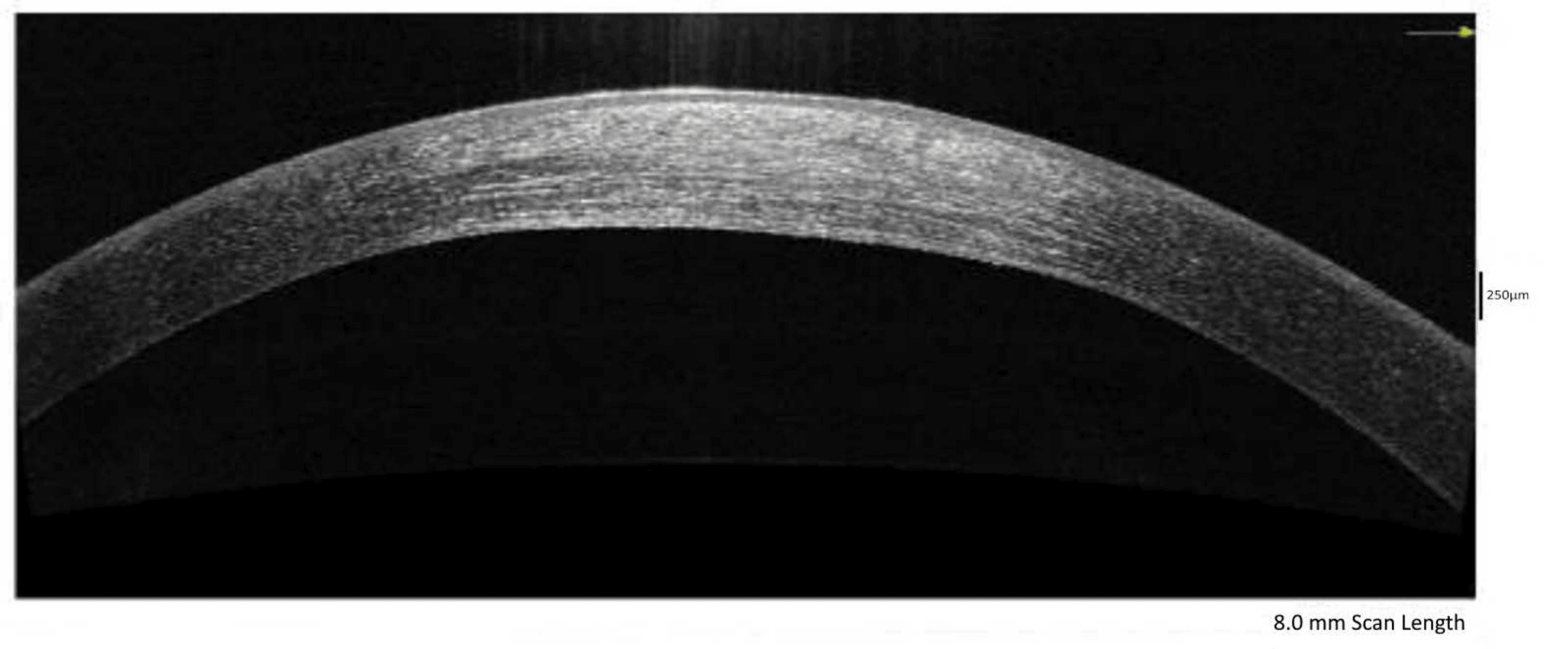
AS-OCT demonstrating resolution of corneal edema and disappearance of endothelial hyperreflective deposits

AS-OCT demonstrated resolution of corneal edema and disappearance of endothelial precipitates (Figure 6).

Repeat IVCM at that time showed Acanthamoeba cysts located deeper within the stroma (approximately 460 μm), while the corneal endothelium appeared normal. Whether this finding represented true migration of cysts or improved visualization of deeper stromal layers following resolution of corneal edema remains uncertain. As the clinical condition stabilized, the frequency of anti-Acanthamoeba treatment, antiviral therapy, and topical corticosteroids was gradually reduced. At the six-month follow-up, BCVA remained 1.0, and IOP was stable. Only mild inferior superficial punctate keratitis persisted. The anterior chamber was quiet, and the corneal endothelium showed mild pigment deposition. IVCM demonstrated no detectable Acanthamoeba cysts at any stromal depth, although central keratocyte irregularities and absence of corneal nerves were noted, consistent with previous infection. Despite the absence of corneal nerves on IVCM, the patient reported no ocular discomfort, and corneal sensitivity was clinically preserved.

Both antiviral and anti-amoebic treatments were further tapered. At the last follow-up examination, 12 months after presentation, no recurrence was observed. The patient had not resumed CL wear and was advised to avoid future water exposure while wearing CLs. Table 1 shows the chronological summary of the patient's clinical course, diagnostic investigations, and treatment.

**Table 1:** Chronological summary of the patient's clinical course, diagnostic investigations, and treatment BCVA: best-corrected visual acuity, PCR: polymerase chain reaction, IVCM: in vivo confocal microscopy, PHMB: polyhexamethylene biguanide, KPs: keratic precipitates, AS-OCT: anterior segment optical coherence tomography, HSV: herpes simplex virus, IOP: intraocular pressure

Time	Clinical findings	Investigations	Treatment
Day 0	Ring infiltrate, epithelial defect, BCVA 0.2	Corneal scraping (negative), PCR (negative), IVCM positive for Acanthamoeba cysts	Topical PHMB and topical chlorhexidine every 30 min; topical voriconazole, fortified antibiotics, atropine twice daily; oral moxifloxacin 400 mg once daily
Days 1-11	Gradual epithelial healing, BCVA improved to 0.5	Clinical follow-up	Continued anti-amoebic therapy
Day 12	Corneal edema, KPs, BCVA decreased to 0.1	AS-OCT, aqueous PCR positive for HSV	Added oral valaciclovir 1000 mg three times daily + topical dexamethasone four times daily
Days 12-22	IOP increased to 28 mmHg	IOP monitoring	Oral acetazolamide 250 mg twice daily
3 months	BCVA 1.0, edema resolved	AS-OCT, IVCM	Gradual tapering
6 months	No active keratitis	IVCM negative for cysts	Continued taper
12 months	No recurrence	Clinical examination	Treatment completed

## Discussion

AK is an uncommon but potentially devastating corneal infection that predominantly affects CL wearers. Exposure of CLs to contaminated water sources, including swimming pools or tap water, represents a well-established risk factor for infection [7]. Although relatively rare compared with bacterial or herpetic keratitis, AK remains clinically important because delayed diagnosis frequently results in a prolonged disease course, significant stromal damage, and permanent visual impairment [8].

To our knowledge, reports of simultaneous AK and HSV endotheliitis are exceedingly rare. Most published cases describe HSV epithelial or stromal keratitis following AK treatment, whereas isolated HSV endotheliitis during active anti-amoebic treatment has been reported only sporadically. This case therefore adds to the limited evidence that secondary herpetic endotheliitis should be considered when new corneal edema, KPs, and visual deterioration develop despite otherwise improving AK.

The pathogenesis of AK is closely related to the organism's biological characteristics. Acanthamoeba exists in two life stages: the metabolically active trophozoite and the dormant cyst. The trophozoite form is responsible for tissue invasion and corneal destruction, whereas the cystic form is highly resistant to environmental stress and pharmacologic therapy. These cysts can persist within the corneal stroma for prolonged periods and are considered a major cause of treatment resistance and recurrence. As the infection progresses, the disease typically evolves from an initial epithelial phase to a deeper stromal inflammatory stage, often associated with the characteristic ring-shaped stromal infiltrate seen in advanced disease [9]. Whether the HSV infection represented reactivation of a latent virus or a newly acquired coincidental infection could not be determined in our patient.

One of the major clinical challenges in AK is its ability to mimic other forms of infectious keratitis, particularly herpetic keratitis. Early manifestations such as epithelial defects, pseudodendrites, or nonspecific stromal infiltrates may resemble HSV infection, frequently leading to misdiagnosis and inappropriate treatment. Although severe ocular pain disproportionate to clinical findings is classically described as a hallmark of AK, this symptom is not universally present, and its absence does not exclude the diagnosis. In the present case, the patient did not report severe pain, illustrating how the clinical presentation may be atypical and potentially misleading [10].

Because of these diagnostic difficulties, adjunctive diagnostic techniques have become increasingly important. IVCM has emerged as a valuable noninvasive modality for detecting Acanthamoeba cysts and trophozoites, with high reported sensitivity and specificity. In our patient, IVCM played a critical role in establishing the diagnosis when initial microbiological testing was negative. The identification of cysts within the anterior stroma allowed early initiation of targeted anti-amoebic therapy, which is essential for disease control [11].

Nevertheless, IVCM has certain limitations, including operator dependency in image acquisition and interpretation, as well as limited availability in many ophthalmic centers, which may restrict its widespread use in routine clinical practice.

Management of AK remains challenging and typically requires prolonged treatment. The cornerstone of therapy is topical biguanides such as PHMB or chlorhexidine, often administered intensively during the initial phase of treatment. These agents are active against both trophozoites and cysts and are usually combined with additional antimicrobial or antifungal agents to broaden antimicrobial coverage and reduce the risk of secondary infection [4]. Despite appropriate therapy, the clinical course is often prolonged due to persistent cysts in the cornea [12].

The mechanisms underlying HSV reactivation during the course of AK remain incompletely understood. Several hypotheses have been proposed. Corneal epithelial disruption and stromal inflammation caused by Acanthamoeba infection may facilitate reactivation of latent HSV within the trigeminal ganglion or corneal tissue. In addition, prolonged local inflammation, alterations in the ocular immune microenvironment, and tissue injury may further promote viral reactivation. Although topical corticosteroids have also been suggested as a potential contributing factor in HSV reactivation, in the present case they were introduced only after clinical suspicion of viral endotheliitis and confirmation of HSV DNA by aqueous humor PCR, making corticosteroid-induced reactivation less likely.

The role of corticosteroids in AK remains controversial and represents a particularly challenging aspect of management. Historically, corticosteroid use was considered detrimental because of concerns that it could suppress host immune responses and promote amoebic proliferation, leading to worsening infection and poorer visual outcomes. Experimental studies and earlier clinical observations suggested that corticosteroid therapy might exacerbate disease severity and increase the likelihood of surgical intervention, including penetrating keratoplasty [13,14].

More recent clinical studies, however, suggest that corticosteroids may be used cautiously in selected cases once effective anti-amoebic therapy has been established. Current evidence indicates that the timing of corticosteroid initiation is critical [15]. In clinical practice, corticosteroids are generally introduced only after evidence of adequate control of AK, including improvement or resolution of epithelial defects, stabilization of stromal infiltration, and ongoing intensive anti-amoebic therapy. Administration of corticosteroids before initiation of anti-amoebic therapy, particularly in cases misdiagnosed as herpetic keratitis, has been associated with severe disease progression. In contrast, delayed introduction of corticosteroids after adequate control of the infection may help reduce stromal inflammation, alleviate pain, and limit corneal scarring without significantly compromising antimicrobial efficacy [16]. Nevertheless, corticosteroid use in AK generally requires prolonged anti-amoebic therapy and careful clinical monitoring.

In our patient, the decision to initiate topical corticosteroids was based on several factors: (1) the patient had already received intensive anti-amoebic therapy for approximately two weeks, with initial improvement and closure of the epithelial defect; (2) new corneal edema and KPs developed, findings more consistent with endothelial inflammation than progressive Acanthamoeba infection; and (3) HSV infection was confirmed by aqueous humor PCR. These findings fulfilled the clinical criteria for HSV endotheliitis, for which corticosteroids, administered under adequate antiviral coverage, represent the standard of care. Importantly, anti-amoebic therapy was continued throughout treatment to minimize the risk of Acanthamoeba recurrence.

The presented case illustrates an even more complex therapeutic dilemma due to the coexistence of HSV endotheliitis, a condition in which topical corticosteroids are considered a cornerstone of treatment. Approximately two weeks after initiation of anti-amoebic therapy, the patient developed corneal edema and KPs suggestive of endothelial inflammation. Aqueous humor PCR confirmed HSV infection, allowing initiation of targeted antiviral therapy. The addition of systemic valaciclovir, together with topical corticosteroids, resulted in rapid clinical improvement, while anti-amoebic therapy was continued to prevent recurrence of the underlying infection [10].

The transient elevation in IOP observed in our patient was most likely attributable to HSV-associated trabeculitis, as it developed at the onset of endotheliitis and prior to prolonged corticosteroid treatment, making a steroid response less likely.

This case report has several limitations. First, baseline HSV serology was unavailable, precluding confirmation of prior HSV exposure. Second, aqueous humor PCR was performed only after the onset of endothelial inflammation; therefore, asymptomatic viral shedding at presentation cannot be excluded. Third, the patient had received topical fluoroquinolone before referral, which may have reduced the sensitivity of microbiological cultures. Finally, although the patient remained recurrence-free during 12 months of follow-up, longer observation would be valuable to assess the long-term risk of recurrent HSV disease or AK.

To our knowledge, reports of concomitant AK and HSV endotheliitis remain exceptionally rare. Most published cases describe epithelial or stromal HSV keratitis, whereas isolated HSV endotheliitis developing during successful anti-amoebic treatment has been reported only sporadically [6,17]. Several mechanisms may explain the coexistence of these pathogens. Corneal epithelial disruption caused by Acanthamoeba infection may facilitate viral entry or reactivation of latent HSV infection within the corneal tissue or trigeminal ganglion [18]. Additionally, prolonged inflammation and local immune dysregulation during AK may predispose the cornea to secondary viral infection. These mechanisms may explain the development of HSV endotheliitis in our patient during treatment.

Recognition of this rare association is clinically important because the therapeutic strategies for the two conditions may appear contradictory. While corticosteroids are traditionally avoided in active AK, they are essential for controlling HSV-mediated endothelial inflammation [19]. In such cases, careful balancing of anti-amoebic and antiviral therapies is required to control both infections simultaneously while minimizing the risk of disease progression [20].

Clinicians should suspect HSV co-infection in patients with AK who initially improve with anti-amoebic therapy but subsequently develop new corneal edema, KPs, or unexpected deterioration in visual acuity, as these findings may represent a change in disease phenotype rather than progression of AK.

## Conclusions

Our case highlights several important clinical points. First, AK should be strongly suspected in CL wearers presenting with keratitis, particularly with a history of water exposure. Second, IVCM can be extremely useful for early diagnosis when conventional microbiological tests are inconclusive. Third, deterioration during treatment should prompt reconsideration of the diagnosis and investigation for possible co-infection. Finally, with careful diagnostic evaluation and tailored therapy, even complex cases involving multiple pathogens can achieve favorable visual outcomes, although long-term surveillance remains important to detect possible recurrence.
